# Cardioinotropic Effects in Subchronic Intoxication of Rats with Lead and/or Cadmium Oxide Nanoparticles

**DOI:** 10.3390/ijms22073466

**Published:** 2021-03-27

**Authors:** Svetlana V. Klinova, Boris A. Katsnelson, Ilzira A. Minigalieva, Oksana P. Gerzen, Alexander A. Balakin, Ruslan V. Lisin, Ksenia A. Butova, Salavat R. Nabiev, Oleg N. Lookin, Leonid B. Katsnelson, Larisa I. Privalova, Daniil A. Kuznetsov, Vladimir Ya. Shur, Ekaterina V. Shishkina, Oleg H. Makeev, Irene E. Valamina, Vladimir G. Panov, Marina P. Sutunkova, Larisa V. Nikitina, Yuri L. Protsenko

**Affiliations:** 1 Yekaterinburg Medical Research Center for Prophylaxis and Health Protection in Industrial Workers, 620014 Yekaterinburg, Russia; klinovasv@ymrc.ru (S.V.K.); Ilzira-Minigalieva@yandex.ru (I.A.M.); privalovaLI@yahoo.com (L.I.P.); iie@ecko.uran.ru (V.G.P.); marinasutunkova@yandex.ru (M.P.S.); 2 Institute of Immunology and Physiology of the Ural Branch of the Russian Academy of Sciences, 620049 Yekaterinburg, Russia; o.p.gerzen@gmail.com (O.P.G.); balakin_a_a@mail.ru (A.A.B.); lisin.ruslan@gmail.com (R.V.L.); butchini@mail.ru (K.A.B.); salavatik2003@gmail.com (S.R.N.); o.lookin@iip.uran.ru (O.N.L.); lbk@efif.uran.ru (L.B.K.); virus_x@mail.ru (D.A.K.); laranikita63@gmail.com (L.V.N.); y.protsenko@iip.uran.ru (Y.L.P.); 3 School of Natural Sciences and Mathematics, The Ural Federal University, 620002 Ekaterinburg, Russia; vladimir.shur@urfu.ru (V.Y.S.); ekaterina.shishkina@urfu.ru (E.V.S.); 4 The Central Research Laboratory, The Ural State Medical University, 620014 Yekaterinburg, Russia; ommt305@mail.ru (O.H.M.); ivalamina@mail.ru (I.E.V.); 5 Institute of Industrial Ecology, The Urals Branch of the Russian Academy of Sciences, 620049 Ekaterinburg, Russia

**Keywords:** nanoparticles, lead, cadmium, cardiotoxicity, combined action, myocardium contractility, trabeculae, papillary muscles, myosin, in vitro motility assay, bioprotectors

## Abstract

Subchronic intoxication was induced in outbred male rats by repeated intraperitoneal injections with lead oxide (PbO) and/or cadmium oxide (CdO) nanoparticles (NPs) 3 times a week during 6 weeks for the purpose of examining its effects on the contractile characteristics of isolated right ventricle trabeculae and papillary muscles in isometric and afterload contractions. Isolated and combined intoxication with these NPs was observed to reduce the mechanical work produced by both types of myocardial preparation. Using the in vitro motility assay, we showed that the sliding velocity of regulated thin filaments drops under both isolated and combined intoxication with CdO–NP and PbO–NP. These results correlate with a shift in the expression of myosin heavy chain (MHC) isoforms towards slowly cycling β–MHC. The type of CdO–NP + PbO–NP combined cardiotoxicity depends on the effect of the toxic impact, the extent of this effect, the ratio of toxicant doses, and the degree of stretching of cardiomyocytes and muscle type studied. Some indices of combined Pb–NP and CdO–NP cardiotoxicity and general toxicity (genotoxicity included) became fully or partly normalized if intoxication developed against background administration of a bioprotective complex.

## 1. Introduction

This paper continues our series of publications dealing with the cardiotoxic effects of subchronic intoxication with lead and cadmium salts on the contractility of isolated trabecular and papillary muscle preparations in the isometric and physiological modes and on rat myocardial contractile proteins [1,2,3,4]. Both lead and cadmium are two of the most common pollutants of the workroom and ambient air associated with metallurgical and some chemical industries being very frequently present together, including in the form of nano-scale particulates. Indeed, cadmium oxide nanoparticles (CdO–NP) and, even more so, lead oxide nanoparticles (PbO–NP) are widely dispersed in the environment, as they are often released during high-temperature technological processes at agglomeration facilities [5], lead smelters [6], and electric steel making plants [7]. PbO–NP, in particular, result from lead vapor oxidation in the presence of air.

In addition, one of the commercially important cadmium species is nanoparticles of cadmium oxide (CdO–NP), which are used in the production of quantum dots for medical diagnostic imaging and for targeting pharmaceutical agents at disease sites [8].

It is known that undissolved NPs can penetrate into a cell and produce a cytotoxic effect by causing oxidative stress and damage to organelles [9]. Both metals are well known as multi-vector systemic toxicants, but given the special medico-social significance of cardiovascular morbidity and mortality in the working age population, it is important to understand what evidence could support the widespread suggestion that occupational and environmental exposures to lead and/or cadmium are an essential risk factor in the growth of this pathology [1,2,3,4,10,11,12,13,14,15,16,17,18,19,20,21,22,23,24,25,26].

Previously, we described subchronic intoxications induced by repeated intraperitoneal injections with lead oxide and/or cadmium oxide nanoparticles (NPs) 3 times a week during 6 weeks. The intoxications proved to be of mild (with PbO–NP) or moderate (with CdO–NP and CdO–NP + PbO–NP) degree [27]. This can be explained by the fact that the statistically significant increase in the toxic metal content of the rat blood in the exposed groups was markedly less than in a previous experiment of similar design involving soluble salts of cadmium and lead [28].

Nevertheless, the development of intoxication was evidenced

(a) in the blood, by reduced hemoglobin and hematocrit, increased proportions of reticulocytes, elevated thrombocytes and thrombocrit, fewer SH–groups (sulfhydryl groups); and,
(b) in the urine, by elevated excretion of the δ-aminolevulinic acid (δ–ALA).

The hematotoxic effects of PbO–NP were found to be more pronounced than those of CdO–NP. At the same time, intoxication with CdO–NP caused a reduction in the level of low-density lipoproteins. The organ that suffered most from exposure to PbO–NP was kidneys (increased mass and proportion of epithelial cells with degenerative changes in tissue imprints of the proximal tubules), while CdO–NP caused the greatest damage to the liver (increased mass and proportion of degenerated hepatocytes and neutrophils). The protein producing function of the liver was impaired in all exposed groups judging by reduced blood albumin and albumin-globulin ratio.

Importantly, all toxic exposures were found to reduce the levels of calcium, endothelin-1 and angiotensin-converting enzyme (ACE) in blood serum, while the natriuretic peptide content was significantly reduced only in response to combined NP exposure. A consequence of changes in the activity and concentration of vasoconstricting and vasodilating factors may be decreased arterial pressure, which we indeed observed to a greater or lesser extent in all experimental groups. The administration of a bioprophylactic complex (BPC) to the animals proved to be beneficial for the vascular system’s regulation too, as evidenced by the recovery of ACE activity, endothelin-1 concentration and natriuretic peptide content [27].

Specifically, the influence of these nanoparticles on the mechanisms of myocardial contractility regulation as a determinant of stroke volume and cardiac output and adaptation to load has not yet been studied sufficiently.

The present study explores the effects of subchronic exposure of rats to CdO–NP and PbO–NP separately or in combination on the mechanisms of length- and frequency-dependent regulation of contractility in isolated myocardial preparations and on the kinetic characteristics of isolated myocardial contractile proteins.

Today, the response surface methodology (RSM) is one of the most important general methods used in the analysis of combined effects produced by mixtures of bioactive substances, including toxic ones. This method enables the potentialities of effective experimental design to be used for approximating a response function. Constructing such approximation requires choosing an analytical model whose parameters would be determined by fitting to experimental data (see Section 4.8).

## 2. Results

### 2.1. Systemic Toxic Effects of Nanoparticles

Our particular concern as a health risk factor is the genotoxic in vivo effect of these metals, particularly in their combination, evidenced by a dose-dependent increase in the DNA fragmentation coefficient in nucleated cells of circulating blood (Table 1).

The morphometric characteristics of the histological preparations (Table 1) showed that NP exposure increased the number of akaryotic hepatocytes in the liver. Partial enhancement of the liver reparative activity is indicated by an increase in binuclear cells, which was found in the PbO–NP group only. In kidneys, glomeruli diameter decreased in the PbO–NP group, while the tubular epithelium suffered the greatest damage from exposure to CdO–NP.

The PbO–NP and CdO–NP + PbO–NP groups displayed a reduction in cardiomyocyte thickness.

**Figure 1:**
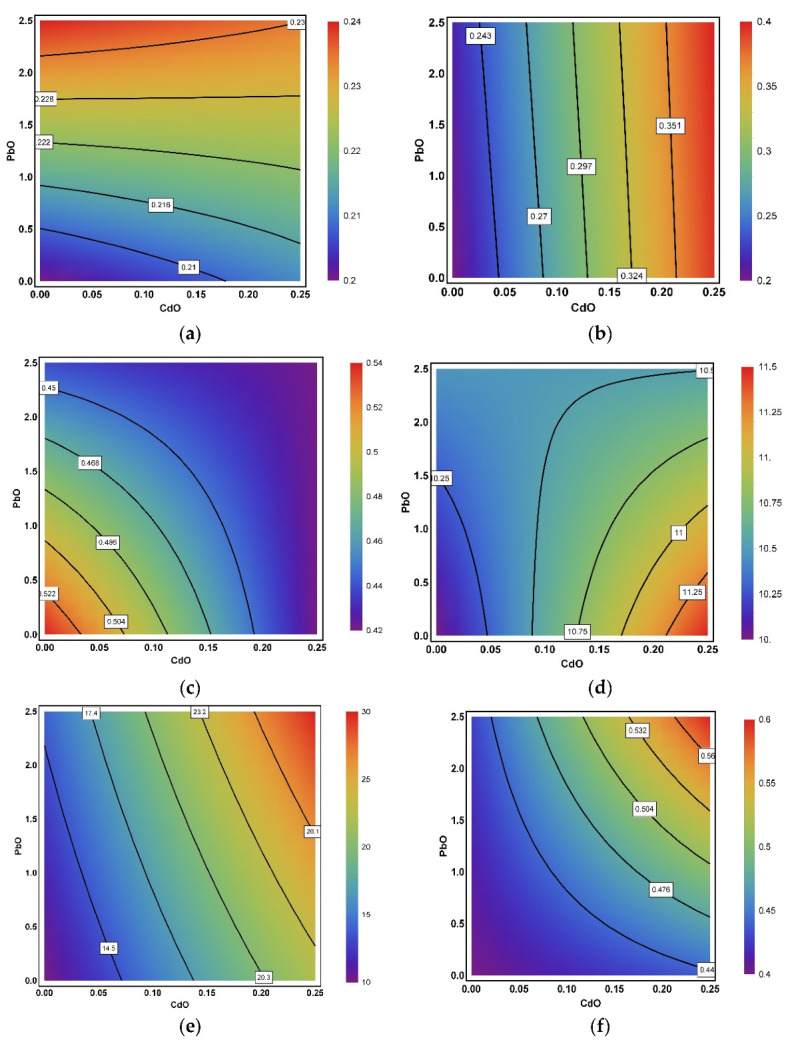
Examples of isobolograms illustrating variations in the type of combined action produced by CdO–NP and PbO–NP on the indices: (**a**) thrombocrit (prevailing single-factor action of PbO–NP, changing to additivity at low doses and to opposite action at high doses), (**b**) eosinophil count (prevailing single-factor action of CdO–NP), (**c**) SH–groups (additivity, changing to subadditivity at high NP doses), (**d**) liver mass (additivity at low NP doses, changing to antagonism at high NP doses), (**e**) acaryotic hepatocytes (additivity), (**f**) coefficient of genomic DNA fragmentation (superadditivity). The axes plot NP doses in mg/kg; numbers on the isoboles show the magnitude of the corresponding effect. The response ranges are distinguished by different colors.

RSM analysis showed that the most prevalent types of general toxic impact produced by CdO–NP and PbO–NP in combination were contradirectionality or the prevailing influence of only one factor in the toxic exposure. We observed all possible types of combined action (Figure 1). It is important to note that such an important index as DNA fragmentation coefficient demonstrated reciprocal enhancement of the harmful effect from NPs (Figure 1f).

The administration of a bioprophylactic complex (BPC) reduced toxic exposure effects judging by the histological data. The kidneys had a lower extent of tubular epithelium desquamation (18.61 ± 4.85% versus 44.28 ± 8.83% in the CdO–NP + PbO–NP group and 0.00 ± 0.00% in the control, *p* < 0.05) and brush border loss (27.49 ± 2.06% versus 57.12 ± 5.69% in the CdO–NP + PbO–NP group and 6.70 ± 1.90% in the control, *p* < 0.05). The liver displayed a lower proportion of acaryotic hepatocytes (20.40 ± 1.28% versus 29.35 ± 2.24% in the CdO–NP + PbO–NP group and 11.38 ± 0.65% in the control, *p* < 0.05). It is important to stress that the DNA fragmentation coefficient equal to 0.5140 ± 0.0025 in the CdO–NP + PbO–NP + BPC group was significantly lower than in the CdO–NP + PbO–NP group (0.5817 ± 0.0016, *p* < 0.05), though being different from the control (0.4123 ± 0.0028, *p* < 0.05).

The BPC also produced a protective effect on cardiomyocyte thickness, which has virtually returned to normal (2.90 ± 0.11 µm versus 2.65 ± 0.08 µm (*p* < 0.05) in the CdO–NP + PbO–NP group, with 3.06 ± 0.09 µm in the control).

### 2.2. Gel Electrophoresis of Rat Myocardium

**Figure 2:**
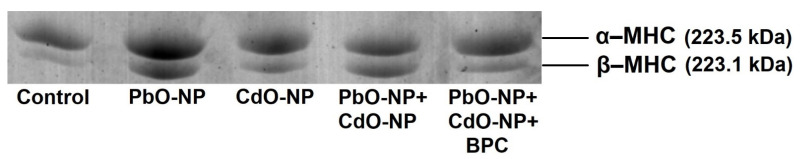
Representative examples of electrophoregrams showing the location of α– and β–myosin heavy chains (MHC) recovered from the myocardium of rats. Left to right: control, group after exposure to PbO–NP, after exposure to CdO–NP, after combined exposure to PbO–NP and CdO–NP, and after combined exposure against oral bioprophylactic complex (BPC) administration.

An electrophoretic study revealed a higher expression of V3 isomyosin, which is a homodimer of β–myosin heavy chains (MHC), in the myocardium of all experimental groups compared with the control (Figure 2, Table 2).

### 2.3. The Contractility of Myocardial Preparations

#### 2.3.1. The Effect of Muscle Length on the Characteristics of Isometric Tension in Isolated Ventricular Trabeculae and Papillary Muscles

In trabeculae, intoxication with CdO–NP and CdO–NP + PbO–NP led to an insignificant growth in active tension, while in papillary muscles NPs reduced its level, and this reduction was statistically significant in response to CdO–NP at large lengths. Changes in active tension under the toxic impact of nanoparticles were insignificant, indicating that the length-dependent regulation of contractions stayed efficient.

**Figure 3:**
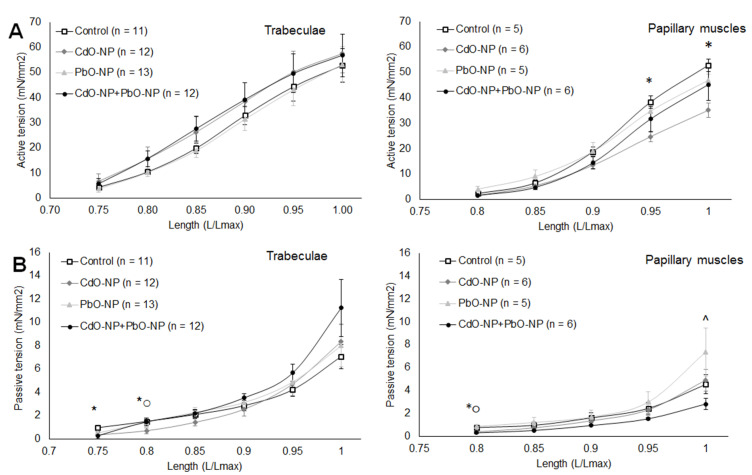
The relationships of the amplitude of active tension (**A**) and passive tension (**B**) vs. relative muscle length, obtained for trabeculae (left panels) and papillary muscles (right panels) of the control, PbO–NP and CdO–NP groups. Muscle length is shown in *L_MAX_* units. Data are shown as mean ± s.e. There are significant differences: *—CdO–NP vs. Control, ^—CdO–NP + PbO–NP vs. PbO–NP, ○—PbO–NP vs. CdO–NP (*p* < 0.05).

We also discovered a small but statistically significant decrease in passive tension (muscle preparation stiffness) in response to CdO–NP at 0.75–0.8 L_MAX_ in both types of muscle (Figure 3). At the same time, the muscle preparations tended to grow stiffer with length. Only in response to a combined action of NPs did the papillary muscles tend to demonstrate lower passive tension.

**Figure 4:**
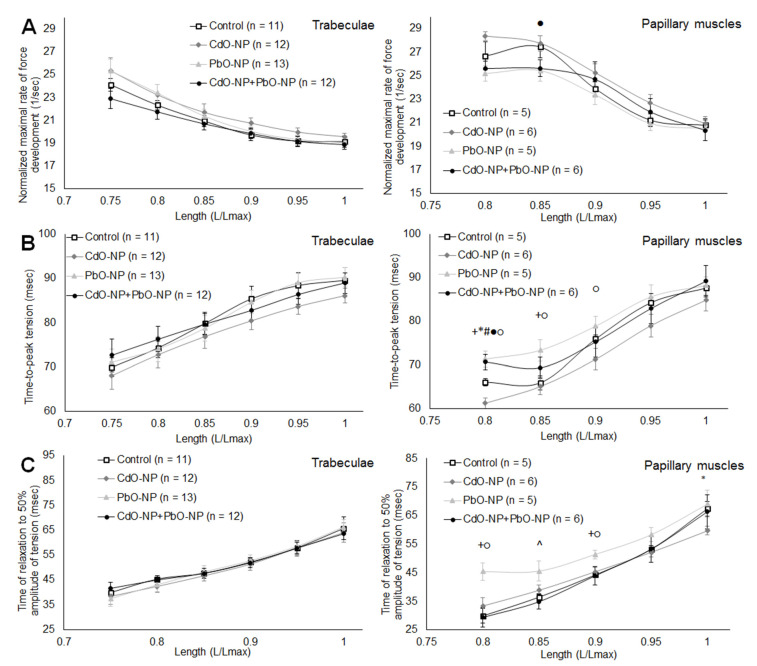
The relationships of the maximal normalized rate (**A**), time-to-peak of force development (**B**) and time of relaxation to 50% amplitude of tension (**C**) vs. relative muscle length, obtained for trabeculae (left panels) and papillary muscles (right panels). Muscle length is shown in *L_MAX_* units. Data are shown as mean ± S.E. There are significant differences: *—CdO–NP vs. Control, +—PbO–NP vs. Control, #—CdO–NP + PbO–NP vs. Control, ●—CdO–NP + PbO–NP vs. CdO–NP, ^—CdO–NP + PbO–NP vs. PbO–NP, ○—PbO–NP vs. CdO–NP (*p* < 0.05).

The maximal rate of tension development in isometric twitch normalized to peak active tension decreased with relative length in the trabeculae and papillary muscles of all groups, with no significant differences between any groups (Figure 4A). Under CdO–NP intoxication, the curve for the maximal normalized rate of tension development is situated above the control for both trabecular and papillary muscles. Under PbO–NP and CdO–NP + PbO–NP intoxications, the location of the curves is varied.

The time-to-peak (TTP) isometric contraction increased with length for both types of muscle (Figure 4B). In papillary muscles at small lengths, TTP was found to increase under PbO–NP and CdO–NP + PbO–NP exposures and decrease in response to CdO–NP. The same trends to a reduction in TTP under CdO–NP and an increase under PbO–NP intoxication were marked in trabeculae as well. In combined intoxication, the opposite actions of lead and cadmium often cancel each other out.

In papillary muscles, the relaxation time to 50% amplitude of tension under exposure to PbO–NP was seen to decrease, while under exposure to CdO–NP it decreased at length equal to *L_MAX_* (Figure 4C).

#### 2.3.2. Physiological Mode of Loading Sequence Simulating Cardiac Cycle Phases

Studies on isolated myocardial preparations under a physiological sequence of loads imitating the cardiac cycle enable intoxication effects of NPs on the force–velocity relationship and work production to be assessed by the characteristics of the tension–length loop.

##### Force–Velocity Relationship of Isolated Trabeculae and Papillary Muscles and In Vitro Motility Assay of Isolated Contractile Proteins

**Figure 5:**
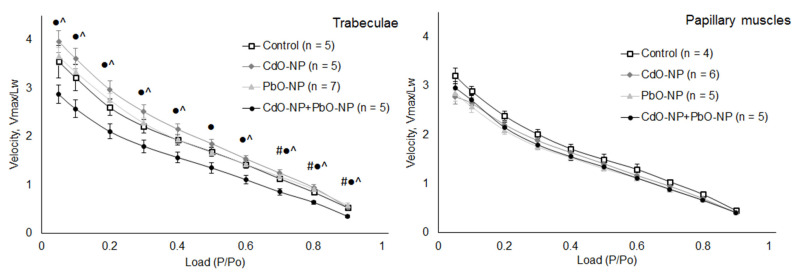
The force–velocity relationships of isolated trabeculae (left panel) and papillary muscles (right panel). Velocity is shown in relative units according to muscle’s working length. Data are shown as mean ± s.e. There are significant differences: #—CdO–NP + PbO–NP vs. Control, ^—CdO–NP + PbO–NP vs. PbO–NP, ●—CdO–NP + PbO–NP vs. CdO–NP (*p* < 0.05).

Both the amplitude and the velocity of muscle shortening depend on the level of afterload, which is reflected by the curve for the dependence of maximal shortening velocity on afterload applied (force–velocity relationship). The force–velocity relationships for trabeculae and papillary muscles of the PbO–NP and CdO–NP groups were typical in shape and non-significantly different from those obtained in the control group (Figure 5). At the same time, combined intoxication caused the force–velocity relationship in trabeculae to decrease statistically significantly at high afterloads (0.7–0.9 P/P_o_). Moreover, the impact of NP combination on trabeculae was different from that produced by PbO–NP and CdO–NP alone over the entire range of afterloads applied.

Since muscle shortening in the absence of load cannot be estimated on multicellular preparations, the velocity characteristics of actin–myosin interaction were assessed in experiments on contractile proteins. An in vitro motility assay allowed us to measure the sliding velocity of a regulated unloaded thin filament over the myosin extracted from the same hearts of rats from all experimental groups. In the ventricular myocardium, we found a prominent decrease in the sliding velocity of unloaded thin filaments on the myosin in both PbO–NP and CdO–NP groups compared to the control (Table 3). The sliding velocity of unloaded thin filaments over myosin decreased less under combined intoxication. Compared with the control, the thin filament sliding velocity over myosin was ~8% less for PbO–NP, ~10% less for CdO–NP, and ~6% less for CdO–NP + PbO–NP.

##### Work Production

**Figure 6:**
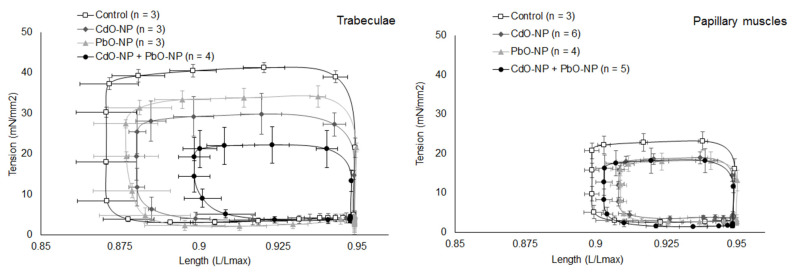
Representative examples of the length–tension loops obtained for isolated trabeculae (left panel) and papillary muscles (right panel). Data are shown as mean ± s.e. Statistical analysis was carried out for work values calculated using the data on length–tension loops.

In all exposed groups, the length–tension loop of the papillary muscles was smaller as compared with that of the trabeculae (compare Figure 6, left and right panels). In other words, the former performed less mechanical work, which corresponds to their function to hold the valves closed during the systole. The amplitude of the length–tension loop, which reflects an increase in the amplitude of mechanical stress in muscles (analogous to isovolumic pressure), was decreased in all experimental groups (Figure 6). Due to this effect, the phase which corresponds to the shortening amplitude (systolic output) was shifted downward relative to the control group.

**Figure 7:**
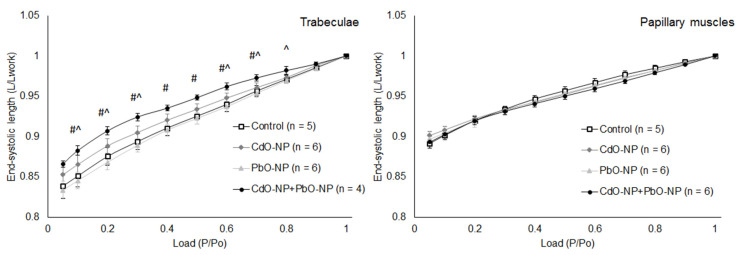
The end–systolic length–afterload relationships of isolated trabeculae (left panel) and papillary muscles (right panel). Data are shown as mean ± s.e. There are significant differences: #—CdO–NP + PbO–NP vs. Control, ^—CdO–NP + PbO–NP vs. PbO–NP NP (*p* < 0.05, Mann–Whitney *U-*test).

For combined intoxication, the amount of shortening normalized to amplitude at 0.95*L_MAX_* was decreased in both types of muscle compared with the control and statistically significantly for trabeculae (Figure 7). It may be noted that the relative shortening amplitude of trabeculae was greater than that of papillary muscles, particularly at small afterloads.

**Figure 8:**
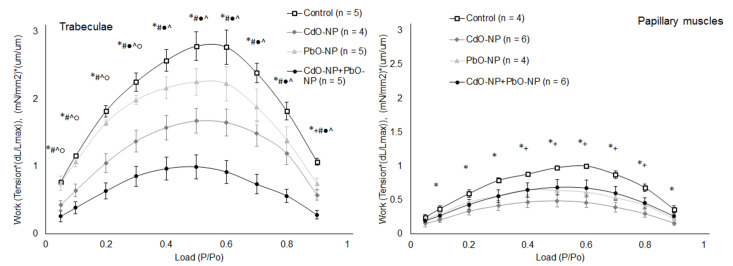
The work–afterload relationships of isolated right ventricular trabeculae (left panel) and papillary muscles (right panel) in the control, PbO–NP, and CdO–NP groups. Data are shown as mean ± S.E. There are significant differences: *—CdO–NP vs. Control, +—PbO–NP vs. Control, #—CdO–NP + PbO–NP vs. Control, ●—CdO–NP + PbO–NP vs. CdO–NP, ^—CdO–NP + PbO–NP vs. PbO–NP, ○—PbO–NP vs. CdO–NP (*p* < 0.05, Mann–Whitney *U*-test).

The relationship between the amounts of mechanical work performed by trabeculae and papillary muscles while contracting against different afterloads was found to be extremal in shape (Figure 8). Note that papillary muscles produced ~2.5 times less mechanical work than trabeculae. In all NP-exposed groups of rats, the amount of work performed by cardiac muscles was found to be decreased. This decrease was statistically significant in trabeculae under CdO–NP and CdO–NP + PbO–NP intoxications, while in papillary muscles, under CdO–NP and PbO–NP exposures.

#### 2.3.3. Post–Rest Potentiation of Contractility

**Figure 9:**
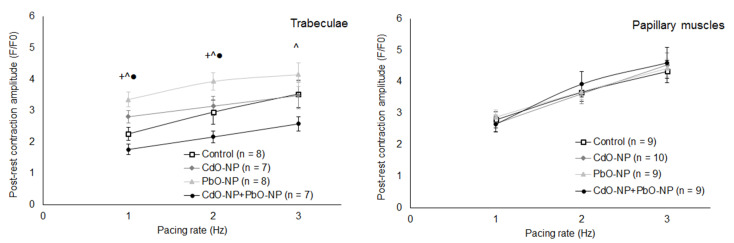
The relationship between post-rest potentiation (assessed as ratio between the amplitudes of the first twitch after 30 s rest and the pre-rest twitch) and pacing rate, obtained for isolated trabeculae (left panel) and papillary muscles (right panel). The pacing rate is varied between 1 and 3 Hz. The muscles are paced at 0.95 *L_MAX_*. Data are shown as mean ± S.E. There are significant differences: +—PbO–NP vs. Control, ●—CdO–NP + PbO–NP vs. CdO–NP, ^—CdO–NP + PbO–NP vs. PbO–NP (*p* < 0.05, Mann–Whitney *U*-test).

Pacing rate variation and, in particular, brief pacing interruptions provide a way to evaluate the influence of intoxication on intracellular Ca^2+^ kinetics and electromechanical coupling in cardiomyocytes [29,30]. Post-rest twitch potentiation—assessed as a ratio between the amplitudes of the first twitch after 30 s rest and the pre-rest twitch—increased in trabeculae and papillary muscles in all groups with increasing the pacing rate from 1 Hz to 3 Hz (Figure 9).

For papillary muscles, this ratio was not changed in any of the experimental and control groups (Figure 9, right panel). For trabeculae under exposure to PbO–NP, the amplitude of the first post-rest twitch was increased compared with the control—statistically significantly at a pacing rate of 1 and 2 Hz. Under combined exposure, on the contrary, it was somewhat reduced compared with the control.

### 2.4. Combined Cardiotoxicity of PbO–NP and CdO–NP

Under exposure to CdO–NP + PbO–NP, it was cadmium that produced the greatest impact on active tension in both muscles and on the end–systolic length in trabeculae (Figure 3). The toxic actions of the NPs were both cancelled out for time-to-peak of force development and time of relaxation to 50% amplitude of tension in papillary muscles and enhanced for the passive tension and the work–afterload relationships in trabeculae, end–systolic length in papillary muscles, and percentage ratio of α– and β–myosin heavy chains. Occasionally, combined exposure produced an effect that was different from that observed for each NP alone (maximal normalized rate, force–velocity relationship and post-rest potentiation in trabeculae).

**Figure 10:**
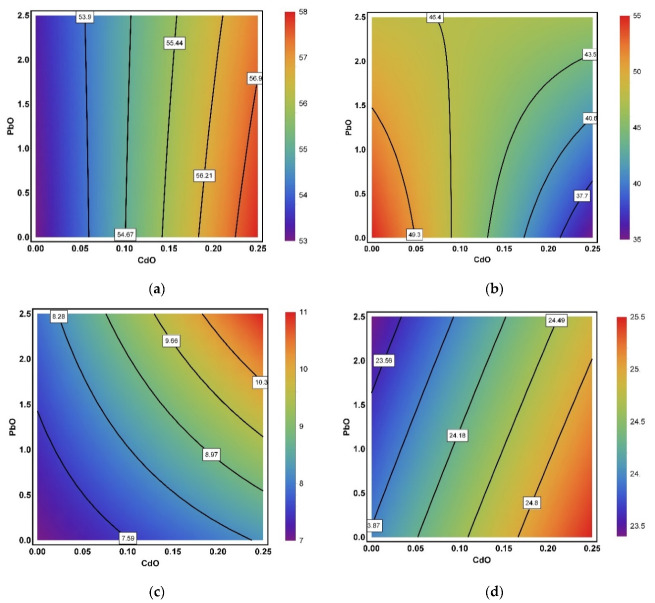

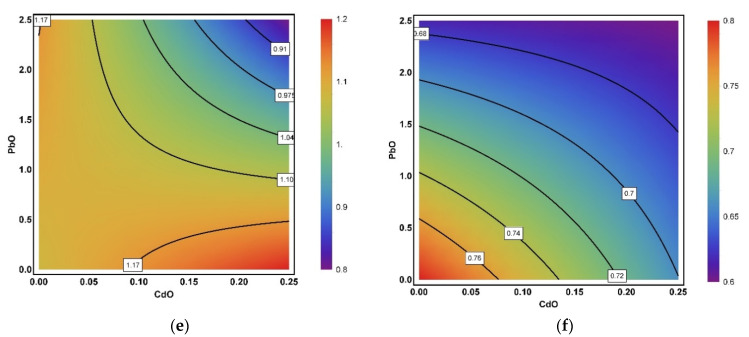
Examples of isobolgrams illustrating variations in the type of combined action produced by CdO and PbO nanoparticles on myocardial contractility: (**a**) active tension in trabeculae at *L = L_MAX_* (single-factor action of cadmium, changing to contradirectionality at high doses), (**b**) active tension in papillary muscles at *L = L_MAX_* (from additivity at low doses through single-factor action of cadmium at low doses of lead to contradirectionality at high NP doses), (**c**) passive tension in trabeculae at *L = L_MAX_* (superadditivity), (**d**) maximal normalized rate in papillary muscles at *L = 0.9L_MAX_* (opposite actions), (**e**) force–velocity relationships in trabeculae at *P = 0.7P_0_* (complex picture from contradirectionality at low doses to additivity at high NP doses), (**f**) maximal shortening velocity of papillary muscles at *P = 0.8P_0_* (additivity, changing to subadditivity). The axes plot doses as fractions of actual value; numbers on the isoboles indicate the level of the corresponding effect).

The picture of CdO–NP and PbO–NP combined action on the contractility of isolated myocardium preparations as confirmed by RSM analysis was varied depending on a range of factors (Figure 10). Besides the specific effect of toxic action, the magnitude of this effect and the ratio of the toxicants involved, the type of combined action was observed to depend also on the degree of cardiomyocyte stretching and the type of muscle investigated.

### 2.5. BPC Administration

A number of rat myocardium mechanical characteristics that suffered change under CdO–NP + PbO–NP intoxication were found to be normalized fully or partially where intoxication developed against background BPC administration (Table 4). The BPC was found to have a beneficial effect on trabeculae in the physiological mode of contraction, normalizing the maximal rate of tension development and end-systolic length.

Moreover, the expression of α– and β–MHC was found to partly normalize under bioprotection. Whereas the sliding velocity of unloaded thin filament on myosin decreased compared with the control under the impact of NP combination without BPC, this decrease was cancelled out in exposure with BPC administration.

As was mentioned above, the combined toxic action of CdO–NP and PbO–NP was attenuated in the presence of the BPC judging by some other effects in various organs and systems as well. It was presented in Table 1 and in the previous paper [27]. We may therefore assume that in relation to cardiotoxicity, this attenuation could be associated not only with a direct action on the myocardium but also with an indirect one through complex inter-systemic relationships.

## 3. Discussion

It has been long known [31,32] that lead and cadmium are nephron- and hepato-toxic poisons. Our study has shown that PbO–NP is more nephrotoxic while CdO–NP is more hepatotoxic, as evidenced by tissue imprints [27] and the majority of histomorphometric data (Table 1). The same was observed for the action of lead and cadmium salts [28].

We should draw attention to the increased DNA fragmentation coefficient found in blood cells in all three toxic exposures and to the superadditive pattern of this effect under combined NP action (Table 1). Previously, we showed that the DNA fragmentation coefficient was also additively enhanced under the combined action of lead and cadmium in ionic form [28].

The reduced activity of angiotensin converting enzyme (ACE) revealed under toxic exposure to PbO–NP and to CdO–NP + PbO–NP could be interpreted as indirect evidence of the lead cardio-vasotoxicity. ACE is produced by epithelial cells in the lungs and heart, with primary localization on the fibroblasts and endothelial vessel cells [33]. The decrease in endothelin-1 content in blood serum could indirectly point to damage to the vasculature as it is synthesized exclusively by endothelial cells [34]. Various mechanisms are involved in the generation of myocardial and vascular responses to endothelin-1 (ET-1) [35]. The attenuation of vasoconstrictor factors (ACE, ET-1), along with cardiomyocyte thinning (Table 1), is most likely to have led to an arterial pressure drop in the experimental groups. In a similar experiment with lead and cadmium salts [28], we observed a different picture: the thickening of cardiomyocytes under the effect of lead and a correlated, although statistically insignificant, increase in arterial pressure, while under cadmium intoxication arterial pressure and cardiomyocyte thickness were decreased. Thus, again, similar biochemical and hemodynamic changes following exposure to CdO–NP and PbO–NP may point to the prevailing contribution of the form of toxic agent rather than its chemical identity.

One of the molecular mechanisms responsible for the toxic action of lead and cadmium on the heart could be their competitive relations with calcium. It has been shown that lead ions effectively substitute for calcium mediating many of calmodulin’s properties [11]. They inhibit calcium–ion binding with troponin C and activate myofibrillar ATPase supported by troponin C [12]. Cd^2+^ participates in a number of Ca^2+^-dependent pathways, attributable to its actions as a Ca^2+^ mimetic, e.g., for calmodulin and the Ca^2+^/calmodulin-dependent proteinkinase II (CaMK–II) that mediates effects on cytoskeletal dynamics and apoptotic cell death [36]. It is characteristic that the diastolic concentration of intracellular calcium in the cardiomyocytes of guinea pigs was found to increase during acute cadmium application (500 nM/L) [37]. This may indicate a decrease in Ca^2+^ uptake by the sarcoplasmic reticulum and/or delayed extrusion through the cellular membrane. Altogether, this may reflect damage to the cellular Ca^2+^ transport systems such as the Na^+^/Ca^2+^ exchanger or Ca^2+^–ATPase.

In our experiments, the decrease in active tension in papillary muscles following toxic NP exposure may be associated with intracellular calcium kinetics. Evidence in favor of this hypothesis is a lowered calcium level in animal blood serum in all toxic exposures, as well as the well-known lead/cadmium antagonism [38,39,40].

It has been shown that the cessation of stimulation for some time before resuming it (to assess post-rest potentiation) may be indicative of the contribution of Ca^2+^ stores in the sarcoplasmic reticulum to the contractile activity [41]. We found that intoxication with PbO–NP significantly increased the post-rest contraction at 1 Hz pacing rate (see Figure 9, left panel). At higher rates, the amplitude of the first post-rest contraction tended to be less elevated in the PbO–NP and CdO–NP groups. At the same time, under combined NP exposure the potentiation of the first post-rest contraction was reduced for all rates studied. This suggests that metal NPs affect the calcium homeostasis in some other way, e.g., by changing the calcium content of the sarcoplasmic reticulum via the modulation of calcium uptake or release and influencing L–type calcium channels [21,25,42]. To elucidate these effects, further experiments with the direct measurement of calcium kinetics in cytosol and/or sarcoplasmic reticulum are needed.

In our study, changes in the rate of isometric tension development after exposure to PbO–NP and CdO–NP were not so much pronounced, but they were directed oppositely. In particular, in papillary muscles following exposure to CdO–NP the rate grew and the TTP decreased, while after exposure to PbO–NP the pattern was reversed. We observed similar effects in the experiment with lead and cadmium ions [3]. In afterloaded contractions, the trabeculae shortening velocity was higher than in the control after exposure to CdO–NP, being significantly different from it after exposure to PbO–NP.

At the same time, in vitro motility assay studies revealed a drop in the velocity of regulated thin filaments after both isolated PbO–NP and CdO–NP and combined NP exposures. These findings correlate with a shift in the expression of myosin heavy chain isoforms towards slower cycling β–MHC (Figure 2). The unambiguous results of the in vitro motility assay, suggestive of the effect of metals on the mechanical activity of cross-bridges, are offset by manifestations of mechanical activity in the intact myocardium—sometimes fully (trabeculae shortening velocity after exposure to PbO–NP), sometimes partially (rate of isometric tension development after exposure to PbO–NP), and sometimes down to the emergence of opposite tendencies (rate of isometric tension development after exposure to CdO–NP). The causes of this oppositely directed pattern observed in an intact myocardium are likely to be associated with certain features in the calcium regulation of contractions and may involve changes in calcium uptake processes in the sarcoplasmic reticulum, the kinetics of calcium-troponin complexes, and competitive relations between metals and calcium within the framework of this kinetics. It should be noted that the decrease in the sliding velocity of thin filaments on myosin in the in vitro motility assay in all groups of toxic NP exposure is different from the results obtained by us in exposure to lead and cadmium salts. In the latter, the sliding velocity was elevated under cadmium and combined exposures [4].

The growth in the end-systolic length with decreasing the tension (which can be seen in the length–tension loops, Figure 6) could be interpreted for the intact heart as a decrease in the pressure created in it and in the end-systolic length (in the pressure–volume loop). This is in agreement with the data showing a decrease in arterial pressure in all experimental groups.

Recently, we reported that rat intoxication with a lead salt solution did not affect the amount of mechanical work performed by both trabeculae and papillary muscles [2]. Meanwhile, now we have found that exposure to PbO–NP led to a drop in the amount of mechanical work produced by papillary muscles. A significant depression of mechanical work performed by both trabeculae and papillary muscles was found under subchronic intoxication with CdO–NP. Work production was lowest in trabeculae under combined exposure to PbO–NP and CdO–NP.

It should be noted that the ratio of the amount of work performed by trabeculae to the work performed by papillary muscles at the afterload ~0.5 P_0_ became greater under NP intoxication. It is known that mechanical loading is one of the main factors of myocardial remodeling [43,44]. It is reasonable to assume that differences between the parietal trabeculae and papillary muscles from the same chamber of the heart are mainly determined by their functions in the cardiac cycle with different loads applied to the muscles. In this regard, one can expect to see different sensitivities to different inotropic factors and intoxications in experimental preparations.

## 4. Materials and Methods

### 4.1. Method of Preparation and Physical and Chemical Characteristics of Lead Oxide (PbO) and Cadmium Oxide (CdO) Nanoparticles

Satisfactorily stable suspensions of nanoparticles of lead oxide (PbO–NP) and cadmium oxide (CdO–NP) were produced by laser ablation of respective 99.99% pure metallic targets under a layer of deionized water. Metal ablation was performed using Fmark-20RL laser material processing system (Laser Technology Center, St. Petersburg, Russia), based on ytterbium-doped pulsed fiber laser (pulse length 100 ns, repetition rate 21 kHz, wavelength 1064 nm). This technique is regularly employed by our team and has been described in sufficient detail more than once (e.g., [9]). It was specially developed by the “Modern Nanotechnologies” Collective Use Center of the Ural Federal University. Scanning electron microscopy revealed that the nanoparticles had a spherical shape and an average diameter of 57 ± 13 nm for CdO–NP and 50 ± 16 nm for PbO–NP. The scanning electron microscope (SEM) CrossBeam Workstation Auriga (Carl Zeiss, Germany) was used for visualization of the nanoparticles.

### 4.2. Experimental Animals

The experiments were carried on 2.5-month-old outbred white male rats from our own breeding colony with the initial body weight of ca. 220 g. The suspension of lead oxide nanoparticles (PbO–NP) at a dose of 2.5 mg/kg of the body weight or cadmium oxide nanoparticles (CdO–NP) at a dose of 0.25 mg/kg of the body weight was intraperitoneally administrated during 6 weeks 3 times a week. Control animals were injected with 2 mL of deionized water. Half of the rats exposed to the NP combination were administered, throughout the exposure period, an assumingly bioprotective complex (BPC) comprising: glutamate (160 mg/rat), cysteine (30 mg/rat), vitamins A (35.2 mkg/rat), E (0.27 mg/rat), C (3 mg/rat), D3 (1,78 mkg/rat), group B vitamins (B1—0.04 mg/rat, B2—0.04 mg/rat, B6—0.04 mg/rat), rutin (1.4 mg/rat), ω–3 polyunsaturated fatty acids (13.3 mg/rat), selenium (1.38 mkg/rat), iodine (4.1 mkg/rat), iron (0.38 mg/rat), calcium (160 mg/rat) and magnesium (2.08 mg/rat) supplements, and pectin enterosorbent (1 g/kg b.w.). Theoretical foundations for the choice and combination of these bioprotectors and their effective use under various intoxications have been considered repeatedly [9,27,28].

The animals were randomly assigned to six groups: Control, PbO–NP, CdO–NP, PbO–NP + CdO–NP, PbO–NP + CdO–NP + BPC and BPC. The rats were housed in conventional conditions, breathed unfiltered air, and were fed standard balanced food. The experiments were planned and implemented in accordance with the “International guiding principles for biomedical research involving animals” developed by the Council for International Organizations of Medical Sciences and the International Council for Laboratory Animal Science (2012) and were approved by the Ethics Committee of the Yekaterinburg Medical Research Center for Prophylaxis and Health Protection in Industrial Workers.

All the clinical laboratory blood and urine tests with the exception of the above ones were performed using the well-known techniques described in many manuals (e.g., [45]).

### 4.3. Random Amplification of Polymorphic DNA (RAPD) Test

For this assay, we totally analyzed 66 blood samples, each sample in three replications. The samples were collected into special vessels cooled to −80 °C. These were then promptly delivered in cryocontainers to a specialized laboratory. To isolate DNA from the cells, we used a GenElute (Sigma) set of reagents in accordance with the manufacturer’s guidelines for use. The DNA content of the samples was determined spectrophotometrically (Ultraspec 1100 pro, Amersham Biosciences, Ltd., Amersham, UK), then they were frozen and stored at −84 °C in a kelvinator (Sanyo Electric Co., Ltd., Moriguchi, Japan) till the beginning of the RAPD (Random Amplified Polymorphic DNA) test performed as described by us elsewhere (e.g., [9]). This technique allows one to quantitatively define the degree of DNA fragmentation as an estimate of the genotoxicity of harmful agents and the protective effects of the bioprotectors studied. The method is based on the fact that, unlike a fragmented DNA, which, in the agarose gel in electrophoresis, forms a so-called comet tail, a non-fragmented DNA has a very low degree of migration and virtually stays in the same place (comet head), the degree of migration being directly related to the degree of DNA fragmentation. DNA amplification was carried out using specific primers and tritiated nucleotides. To characterize the degree of damage to DNA, we used the “coefficient of fragmentation”, i.e., the ratio of total radioactivity of all tail fractions to that of the head.

### 4.4. Histopathology and Morphometry

Liver, kidney and heart tissue sections were prepared from four rats in each treated and control group for microscopic histological examination by the hematoxylin and eosin stain and, where necessary, by periodic acid - schiff (PAS), Nissl or Perl’s stains. For morphometric characterization of these tissues, we used the Avtandilov’s planimetric ocular grid and the image recognition programmed system CellSens (Olympus, Hamburg, Germany).

### 4.5. Determination of α– and β–Cardiac Myosin Heavy Chain (MHC) Ratio

The ratio of myosin heavy chain isoforms in the rat myocardium was determined by denaturing gel electrophoresis (SDS–PAGE). Following the electrophoresis, the gels were Coumassie stained, and, after washing with destaining solution and water, scanned by a GS-800 Calibrated Densitometer (BioRad, Dallas, TX 75243, USA). The percentage ratio of α– and β–MHC was determined in the samples by Image Lab 5.2.1.

### 4.6. In Vitro Motility Assay for Assessment of Mechanical Characteristics of Actin–Myosin Interaction

The effects of intoxication with lead and/or cadmium salts on the characteristics of actin–myosin interaction were studied by means of an in vitro motility assay which we had already used previously [3,4]. In this assay, a fluorescently labeled regulated thin filament (including actin, tropomyosin, and troponin) moves in the presence of ATP and calcium ions (*pC*a = 4) in a flow cell on a surface coated with rat myosin isolated from the same hearts from which the trabecules and papillary muscles were excised, and one can assess actin–myosin mechanical interaction by the characteristics of how thin filaments move. Actin was obtained from rabbit skeletal muscle according to standard procedure [46]. Cardiac troponin was isolated from the left ventricle of a swine heart as described by [47]. Recombinant tropomyosin was obtained as described by [48]. Such usage of contractile and regulatory proteins extracted from animals of different species and combined in a motility assay is a common practice [49,50,51]. Thin filaments were constructed from actin, troponin, and tropomyosin by mixing these proteins in the following concentrations: 400 nM rhodamine–phalloidin labeled F–actin, 100 nM troponin, and 100 nM tropomyosin at 4 °C in the buffer (25 mM KCl, 25 mM imidazole, 4 mM MgCl_2_, 1 mM EGTA (egtazic acid), and 10 mM DTT (Dithiothreitol), pH 7.5). The protein ratio in the thin filaments was checked by 10% SDS–PAGE (sodium dodecyl sulphate–polyacrylamide gel electrophoresis) [52].

The in vitro motility assay was performed as described previously [53]. All experiments were carried out at a temperature of 32 °C. Fluorescently labeled thin filaments were visualized by an Axiovert 200 inverted epifluorescence microscope equipped with a 100×/1.45 Oil alpha Plan–Fluar objective (Carl Zeiss, Germany) and an EMCCD iXon–897BV camera (Andor Technology, USA). Ten fields of 30 s each were usually recorded in every flow cell. Data were analyzed using GMimPro software [54] and the velocities of >100 individual filaments were averaged to determine the mean value ± standard error of mean.

### 4.7. Measurement of the Contractile Response of Isolated Myocardial Preparations

Before killing the rats by cervical dislocation, the animals of all three groups were administered heparin (1000 ME, 0.25 mL per animal). The heart was removed immediately upon euthanasia and placed for 15 min into Krebs–Henseleit saline with 2.3–butanedione monoxime (30 mM). Thin trabeculae and papillary muscles were dissected from the right ventricle of the same heart. The preparations were fixed to the two rods of the length servomotor and force transducer in a thermo-controlled bath (Muscle Research System, Scientific Instruments GmbH, Heidelberg, Germany).

The experiments were performed in a modified Krebs–Henseleit solution (in mM: NaCl 118.5; NaHCO_3_ 14.5; KCl 4.2; KH_2_PO_4_ 1.2; MgSO_4_ 1.2; glucose 11.1, CaCl_2_ 1.9) oxygenated by a mixture of 95% O_2_ and 5% CO_2_, pH = 7.4 at 35 °C. The solution was pumped through a thermo-controlled bath (volume of 5 mL) using a peristaltic pump at a speed of 5 mL/min. The parietal trabeculae and papillary muscles from the same right ventricle were electrically paced by non-polarizing carbon electrodes at 2 Hz with ~5 ms super-threshold rectangular stimuli. All measurements were performed under the following conditions: pacing frequency 2 Hz, temperature 35 °C, and working length 0.95 *L_MAX_*.

The mechanical response under either isometric or physiological mode of contraction was measured using an analogue-to-digital and digital-to-analog converter (PCI–1716S, AdLink Technology Inc., Taiwan) at a frequency of 10 kHz. In the physiological mode, a sequence of loads similar to the physiological sequence in the cardiac cycle was applied to the muscle, which allowed measuring force–velocity relationships (under different afterloads) and force–shortening loops (which resemble the pressure–volume loop typical of the whole heart) [2,4,55,56]. In the biomechanical experiment, the muscle length was increased stepwise to determine the value at which the muscle developed a maximal active component of isometric force (*L_MAX_*). This value was determined individually for each of the preparations and then used as a reference point for analyzing their contractility. For comparing the parameters of mechanical activity between myocardial preparations of different thickness from different rats, the force developed by each muscle was normalized to its cross-sectional area calculated for the central part of the muscle using the measurements of its larger and smaller diameters with a binocular stereoscopic microscope. The calculation assumed that muscles were ellipsoidal in shape. Force was normalized to the estimated cross-sectional area to obtain mechanical tension values. Our approach is different from that employed by other researchers [15,21], who normalized active force to the muscle’s weight. A limitation of this normalizing method is that preparations of the same mass may have different diameters, and it requires careful determination of the mass of the muscle’s parts that are used for attachment and, therefore, excluded from the generation of active force. To estimate force development and relaxation rates, the time course of isometric contraction was normalized to its amplitude.

### 4.8. Mathematical Processing and Analysis

The statistical significance of the inter-group differences between the mean values of all obtained indices was estimated by Student’s *t*-test with a correction for multiple testing (ANOVA test) or Mann–Whitney *U*-test for mechanical characteristics. A Wilcoxon matched pairs test was used to compare dependent samples. The results are presented as mean ± s.e., with differences considered to be statistically significant at *p* < 0.05.

As well as in our previous works on combined toxicity (e.g., [9,57]) and in a recent work by [58] on magnetite nanocrystal clusters, we performed the mathematical modeling of the combined Cd–Pb action by the Response Surface Methodology (RSM) [27,59,60].

The regression equation describing the response surface Y = Y (x1, x2) in our case is:

(1) $$Y\;=\;b_{0}+\;b_{1}x_{1}+\;b_{2}x_{2}+\;b_{12}x_{1}x_{2}$$

where *Y* is a toxicity index, and *x*_1_ and *x*_2_ are the doses of the agents participating in the combination (cadmium and lead, respectively). This equation was constructed by fitting the coefficients *b*_0_, *b*_1_, *b*_2_ and *b*_12_ to experimental data using the ordinary least squares method. It is inferred that two agents produce a unidirectional effect on response *Y* if both one-way response functions *Y* (*x*_1_, 0) and *Y* (0, *x*_2_) either increase or decrease with an increase in *x*_1_ or *x*_2_; on the contrary, two agents are assumed to be acting contra-directionally if one function increases while the other decreases. According to the response surface approach, even in the case of two-level agents, Equation (1) enables one to predict the magnitude of response y for any combination of agent doses within the experimental range for each of them rather than at two points only. When quasi-sectioning the response surface on different levels corresponding to different meanings of the outcome Y, one gets a family of Loewe isoboles which may have the same or a different form and unidirectional or contradirectional slopes, and thus render assigning the types of binary combined toxicity both easy and demonstrative.

There is the only nonlinear polynomial model that can be used in the conducted experiment if the common orthogonal encoding is used for exposure levels. In particular, no quadratic model can be applied to the data. The usual magnitude of the determination coefficient R^2^ for Equation (1) was no less than 0.5.

## 5. Conclusions

1. The moderate multi-organ toxic effects of the subchronic exposure of male rats to CdO–NP and/or PbO–NP have been demonstrated by a number of hematological, biochemical, and histological indices. Many of these effects are similar to those revealed previously for intoxications with Cd and/or Pb soluble salts, while some effects seem to be rather specific to the nano-particulate state as such.
2. We are the first to have obtained phenomenological data for the influence of CdO–NP and/or PbO–NP exposure on the mechanical activity parameters of isolated ventricular trabeculae and papillary muscles, and for the sliding velocity of isolated myofilaments in an in vitro motility assay. It is shown that the sliding velocity falls under exposure to CdO–NP and PbO–NP, both alone and in combination, which is explained by a shift in the expression of myosin heavy chain isoforms towards slower cycling β–MHC.
3. The stability of the tension–length and force–velocity slopes in isolated cardiac muscles from intoxicated rats confirms that under moderate subchronic intoxication with CdO–NP and/or PbO–NP, the heterometric regulation of myocardial contractility is preserved.
4. The toxic impact of metal NPs also manifested itself in decreased mechanical work produced by isolated muscle preparations after the exposure of rats to CdO–NP and PbO–NP, which was more pronounced under their combined action.
5. The type of combined CdO–NP and PbO–NP cardiotoxicity, if modeled with the help of the Response Surface Methodology, appears to be variable depending on a number of factors, in particular on whether it is examined in trabeculae or papillary muscles.
6. Some characteristics of rat myocardium altered by the impact of CdO–NP and PbO–NP intoxication were fully or partially normalized if intoxication developed against background administration of the proposed bioprotective complex (BPC).
7. We believe that this sum of knowledge strengthens the scientific foundations of risk assessment and risk management projects in occupational and environmental settings characterized by human exposure to lead and/or cadmium nanoparticles and may be clinically relevant too.

## Figures and Tables

**Table 1:** Genomic DNA fragmentation coefficient in circulating blood nucleated cells and some morphometric indices of the heart, kidney and liver status in rats exposed to lead oxide (PbO–NP) and/or cadmium oxide (CdO–NP) nanoparticles ($\bar{X}$ ± s.e.).

Index	Groups of Rats Exposed to:			
	Water (Control)	CdO–NP	PbO–NP	CdO–NP + PbO–NP
**Blood nucleated cells**				
Coefficient of genomic DNA fragmentation	0.4123 ± 0.0028	0.4452 ± 0.0004 *	0.4358 ± 0.0003 *●	0.5817 ± 0.0016 *●#
**Heart**				
Right ventricular cardiomyocyte thickness, µm	3.06 ± 0.09	3.07± 0.09	2.80 ± 0.07 *	2.65 ± 0.08 *●
**Kidneys**				
Glomeruli diameter, µm	40.08 ± 1.09	42.65 ± 1.75	32.90 ± 1.01 *●	42.43 ± 0.98 *#*
Epithelial desquamation, %	0.0 ± 0.0	63.06 ± 12.09 *	21.73 ± 5.51 *●	44.28 ± 8.83 *#
Brush border loss, %	6.70 ± 1.90	45.18 ± 5.03 *	33.54 ± 5.44 *	57.12 ± 5.69 *#
Liver				
Akaryotic hepatocytes, %	11.38 ± 0.65	22.30 ± 1.79 *	14.95 ± 0.90 *●	29.35 ± 2.24 *●#
Binuclear hepatocytes, %	3.82 ± 0.43	3.20 ± 0.67	5.35 ± 0.53 *●	2.85 ± 0.42 *#*
Kupffer cells, %	10.55 ± 0.36	24.40 ± 1.00 *	23.75 ± 1.29 *	20.45 ± 1.20 *●

Notes: The superscripts designate values which are statistically significantly different from the corresponding values for the control group (*), for the “CdO–NP” (●) or for the “PbO–NP” (#) (*p* < 0.05 by Student’s *t*–test).

**Table 2:** Percentage of α– and β–myosin heavy chains in myocardium. ($\bar{X}$ ± s.e.).

%	Control	PbO–NP	CdO–NP	PbO–NP + CdO–NP
α–MHC	92 ± 1.06	76 ± 2.04 *	78 ± 1.63 *	61 ± 1.22 *
β–MHC	8 ± 1.06	24 ± 2.04 *	22 ± 1.63 *	39 ± 1.22 *

*—significant difference in comparison with the control group (*p <* 0.05, Mann–Whitney *U-*test).

**Table 3:** The velocities of regulated thin filament sliding movement over rat ventricular myosin in all experimental groups ($\bar{X}$ ± s.e.).

V_MAX_ (µm/s)			
Control	PbO–NP	CdO–NP	PbO–NP + CdO–NP
4.90 ± 0.05	4.60 ± 0.05 *	4.45 ± 0.10 *	4.63 ± 0.09 *

*—significant differences in comparison with the control group (*p <* 0.05, Mann–Whitney *U-*test).

**Table 4:** Some characteristics of the myocardium in control rats and in those exposed to lead and cadmium oxide NP combination with and without bioprotection ($\bar{X}$ ± s.e.).

Myocardium Characteristics	Group of Rats		
	Controls	Exposed to CdO–NP + PbO–NP	Exposed to CdO–NP + PbO–NP along with BPC
Expression of α–MHC, %	92 ± 3	61 ± 3 *	85 ± 2 *▪
Expression of β–MHC, %	8 ± 3	39 ± 3 *	15 ± 3 *▪
Sliding velocity of thin filaments over myosin (*V_MAX_*), µm/s	4.90 ± 0.15	4.63 ± 0.22 *	4.81 ± 0.27 ▪
Velocity of trabeculae at working length corresponding to 0.95 *L_MAX_* and load corresponding to 0.9 *P/P_0_*, *V_MAX_/Lw*	0.53 ± 0.03	0.35 ± 0.03 *	0.54 ± 0.03 ▪
End–systolic length of trabeculae at working length corresponding to 0.95 *L_MAX_* and load corresponding to 0.6 *P/P_0_*, *L/Lwork*	0.94 ± 0.01	0.96 ± 0.00*	0.95 ± 0.01 ▪

Note: *—statistically significant difference from the control group, ▪—statistically significant difference from the group exposed to NPs (*p* < 0.05, Mann–Whitney *U*-test).

## Data Availability

The data presented in this study are available on request from the corresponding author.
